# EEG Based Decoding of the Perception and Regulation of Taboo Words

**DOI:** 10.1111/psyp.70318

**Published:** 2026-05-18

**Authors:** Parisa Ahmadi Ghomroudi, Michele Scaltritti, Bianca Monachesi, Atefeh Jalali, Peera Wongupparaj, Remo Job, Alessandro Grecucci

**Affiliations:** ^1^ DiPSCo – Department of Psychology and Cognitive Sciences University of Trento Rovereto Italy; ^2^ International School for Advanced Studies – SISSA Trieste Italy; ^3^ Faculty of Psychology Chulalongkorn University Bangkok Thailand; ^4^ Department of Education, Psychology and Communication Sciences University of Bari Aldo Moro Bari Italy

**Keywords:** acceptance, emotion regulation, emotional words, event related potential, late positive potential, support vector machine

## Abstract

Emotional meaning is often conveyed through language, and certain word categories, such as taboo words, can elicit strong affective responses that may require regulation. While taboo words are socially and psychologically salient, their neural processing remains to be fully explored. In this study, we investigated whether EEG signals can be used to predict neutral, negative, and taboo words, and whether this prediction is preserved under conditions of emotion regulation. Forty native Italian speakers viewed 240 words across these categories while EEG was recorded. Participants completed two conditions: a *Look* condition (passive observation) and an *Accept* condition (emotion regulation via acceptance). Using support vector machine (SVM) classifiers applied to event‐related potentials (ERPs), we found that word categories could be reliably decoded from the late positive potential (LPP) in central‐parietal‐occipital and anterior right regions between 450 and 850 ms. Notably, classification remained above chance but largely reduced even during the Accept condition, suggesting that word‐related affective information persists at the neural level despite the regulation effort. These findings advance our understanding of emotional language processing and highlight the utility of machine learning for decoding subtle neural representations.

## Impact Statement

This study advances the understanding of emotional language by applying Support Vector Machine (SVM) decoding to EEG data on taboo word processing. We demonstrate that taboo words elicit distinct neural signatures, particularly within the Late Positive Potential window, which remain detectable even during emotion regulation. These findings offer novel insights into the robust neural encoding of taboo connotation and highlight the utility of multivariate pattern analysis for investigating complex emotional dynamics.

## 1 Introduction

In everyday interactions, emotions are often communicated and elicited through language. This is especially evident in psychotherapy, where words serve as the primary tool for expressing, elaborating, and regulating emotional experiences for both clients and therapists (Grecucci et al. 2019). Among emotionally charged words, taboo words constitute a particularly salient category due to their distinct psychological and social implications. Swearing has been shown to trigger strong emotional reactions (Sheidlower 2009), enhance the impact of communication (Azzaro 2018), elicit humor (Blake 2018), and even help regulate emotions and alleviate physical pain (Stephens and Umland 2011). Moreover, taboo language can increase the persuasive power of messages (Cavazza and Guidetti 2014), highlighting its functional relevance in both interpersonal and clinical contexts.

Taboo words are considered a distinct class of linguistic stimuli with both emotional and social dimensions. They are typically high in arousal and low in valence, reflecting their strong emotional impact and attention‐capturing properties (Jay 2009; Reilly et al. 2020). Beyond affective intensity, taboo words also convey socially inappropriate or norm‐violating content, carrying context‐dependent social and pragmatic meaning that distinguishes them from other negative words (Frazier et al. 2014, 2018; Wabnitz et al. 2012). Accordingly, taboo words can be conceptualized as social–affective cues that simultaneously engage emotional processing and representations of social norms, rather than as purely negative emotional stimuli. In this perspective, taboo words are not merely extreme versions of emotional language, but rather distinct entities requiring an expanded, three‐dimensional framework. While traditional models of emotional word processing rely primarily on valence and arousal, this framework introduces social tabooness or pragmatic content as a third dimension (Donahoo and Lai 2020). This additional dimension captures the extent to which taboo words function as socially salient signals of norm violation, engaging evaluative processes related to social appropriateness and moral meaning.

Emotional word processing is characterized by a well‐established cascade of ERP responses. Emotional words elicit an early posterior negativity (EPN) around 200–300 ms over left occipito‐temporal sites, reflecting rapid and largely automatic prioritization of emotionally salient word meaning following lexical access, most commonly driven by arousal. This early stage is followed by a late centro‐parietal positive potential/complex (LPP/LPC), typically observed between ~400 and 650 ms, which reflects more elaborative, attention‐ and task‐dependent evaluative processing (Citron 2012; Schupp et al. 2000). Together, these components index successive stages of affective and motivational processing in written language and provide a reference framework for investigating how specific classes of emotionally and socially salient words, such as taboo words, may engage these mechanisms (Kissler et al. 2007, 2009; Schacht and Sommer 2009; Palazova et al. 2011; Kissler and Herbert 2013; Bayer and Schacht 2014). Emotional words, both positive and negative, have been shown to elicit stronger LPP responses than neutral ones, often extending into frontal regions over time (Citron 2012; Frühholz et al. 2011; Herbert et al. 2008; Hinojosa et al. 2010; Hofmann et al. 2009; Kanske and Kotz 2007; Palazova et al. 2011; Zhang et al. 2014). Some studies have also reported a negativity bias, where negative words evoke larger or more sustained LPPs than positive ones (e.g., Hajcak and Olvet 2008; Ito et al. 1998). Recent ERP evidence, however, suggests that taboo words cannot be fully understood within traditional emotion‐word frameworks. While taboo and negative words elicit comparable positivity effects in an earlier time window (~250–550 ms), consistent with attention to negative emotional salience, taboo words show a sustained and enhanced late positivity in the LPP (Donahoo and Lai 2020). This late effect has been interpreted as reflecting evaluative processes related to social norms and pragmatic appropriateness, rather than differences in arousal or valence per se. It remains unclear whether the difference between taboo and neutral or negative words lies entirely in this neurophysiological marker.

The first aim of this study is to investigate the psychological and neural responses elicited by taboo words in comparison to negative and neutral words. Specifically, we test whether ERP responses, particularly the LPP, carry enough information to differentiate these word categories. To this end, we employ a machine learning method, namely SVM, which has been shown to detect distributed neural patterns that traditional univariate analyses may overlook (Grootswagers et al. 2017; Ritchie et al. 2015).

A second question concerns how these responses are modulated by emotion regulation. In daily life, we often modulate our affective reactions to emotionally salient stimuli, including language, in order to conform to social norms. Emotion regulation plays a vital role in psychological well‐being (Gross and Levenson 1993) and is a central component of many psychotherapeutic approaches (Beck et al. 1979; Grecucci et al. 2019; Hayes 2004). Deficits in emotion regulation are implicated in various clinical disorders (Joormann and Gotlib 2010; Nolen‐Hoeksema et al. 2008). The LPP has been found to reflect the intentional engagement with emotional content and is thus a key neural marker for studying regulation strategies (Fields and Kuperberg 2016; Foti et al. 2009; Hajcak et al. 2010). While most ERP studies have examined the effects of reappraisal on emotional pictures, fewer have explored the regulation of emotional words. Prior works have shown mixed effects for different strategies and stimuli. For example, Grecucci et al. (2019) found that distancing enhanced brain activity for unpleasant pictures but not for words, while Baker et al. (2017) reported strategy‐specific effects on unpleasant word processing. In the present study, we focus on a less‐studied strategy, acceptance, which involves adopting an open, non‐judgmental stance toward emotional experiences. Acceptance has been associated with reductions in emotional reactivity and physiological responses (Campbell‐Sills et al. 2006; Hofmann et al. 2009). However, whether this strategy alters the electrophysiological correlates of emotional words processing remains unclear.

Thus, the second aim of our study is to examine whether affective word categories can still be distinguished at the neural level when acceptance is used to regulate emotions. We anticipated that acceptance—a strategy involving open, non‐judgmental observation of emotional responses—may reduce elaborative evaluative processing, and this would be reflected in attenuated LPP amplitudes (Citron 2012). Using machine learning (ML), we test whether distributed neural patterns still contain enough information to distinguish between taboo, negative, and neutral words. This allows us to assess not only the sensitivity of MVPA but also the nature of emotion regulation itself: whether acceptance merely dampens neural responses or fundamentally transforms how emotional meaning is encoded in the brain.

Finally, a third aim of our study is to identify any event‐related responses associated with the implementation of the acceptance strategy during the presentation of the stimuli. While reappraisal and distancing are linked to consistent, top‐down cortical modulations (e.g., stimulus‐preceding negativity; Grecucci et al. 2019), acceptance may involve more bottom‐up, experiential processes that lead to subtler or more diffuse neural changes (Messina et al. 2021; Monachesi et al. 2023). Since EEG primarily captures cortical activity, we hypothesize that a classifier trained to distinguish between the Accept and Look conditions may not achieve high accuracy, suggesting that acceptance may not produce consistent cortical signatures detectable at the scalp level via EEG, and more subcortical activity.

To address these questions, participants viewed 240 emotional words (neutral, negative, taboo) while EEG was recorded. They performed two tasks: Look (passive observation) and Accept (emotion regulation via acceptance). ERP responses were analyzed using both traditional univariate methods and SVM‐based classification. While cluster‐based permutation tests assess statistically significant amplitude differences across electrodes and time points (Maris and Oostenveld 2007), ML captures distributed spatiotemporal patterns across trials, offering higher sensitivity to subtle condition‐specific neural representations (Grootswagers et al. 2017; Donos et al. 2022).

## 2 Methods

### 2.1 Participants

Forty native Italian speakers (16 males; mean age = 23.78 years, SD = 3.40; mean years of education = 15.64, SD = 2.01) participated in the study. All participants were right‐handed with normal or corrected‐to‐normal vision, no auditory impairments, and no reported neurological, psychiatric, or learning disabilities. Although no formal a priori power analysis was conducted, the sample size was determined based on standards from prior ERP studies investigating emotion regulation using word stimuli (e.g., Grecucci et al. 2019). Three participants were excluded due to the excessive number of noisy epochs (> 25%), and two were removed due to artifacts induced by a malfunctioning EEG cap, leaving 35 participants in the final sample. All participants signed an informed consent document and were compensated with €15 for about 3 h of involvement, including preparation and experimental procedures. The study procedure received approval from the University of Trento research ethics committee, protocol number (2021–033). All procedures were conducted in accordance with the Declaration of Helsinki.

### 2.2 Stimuli

Eighty neutral and 80 negative words were selected from the Italian adaptation of the Affective Norms for English Words (ANEW; Bradley and Lang 1999; Montefinese et al. 2014). Additionally, 80 taboo words were sourced from the Italian Taboo Words database (ITABOO; Sulpizio et al. 2020). Words in the three categories were comparable with respect to the psycholinguistic variables shown in Table 1. For purposes of counterbalancing, each category of stimuli was split into two subsets, each containing 120 items.

**TABLE 1:** Mean psycholinguistic variables of the stimuli used in the experiment.

Stimulus type	Length of letter	Orthographic neighborhood	Familiarity	Concreteness	Valence	Arousal	Log of frequency
Neutral	7.30 ± 1.93	3.81 ± 5.32	5.84 ± 0.96	6.16 ± 1.70	5.69 ± 0.71	4.76 ± 0.68	6.04 ± 1.84
Negative	7.56 ± 1.87	3.29 ± 4.30	5.58 ± 1.03	6.03 ± 1.30	2.19 ± 0.38	6.32 ± 0.57	6.13 ± 1.76
Taboo	7.44 ± 1.79	4.81 ± 8.03	5.74 ± 1.16	6.46 ± 1.27	4.25 ± 1.03	5.01 ± 0.58	5.74 ± 1.89

*Note:* Psycholinguistic and affective ratings.

### 2.3 Apparatus and Procedure

#### 2.3.1 Training

Before EEG recording, participants completed a brief training session to standardize the use of the acceptance‐based emotion regulation strategy and clarify its implementation. The experimenter provided both verbal and written instructions to ensure a clear understanding of the strategy. To practice the strategy in a controlled context, participants were shown 10 unpleasant images drawn from the International Affective Picture System (IAPS) (Lang et al. 2008). These images were selected to evoke negative emotional responses, allowing participants to rehearse the acceptance strategy under conditions comparable to the experimental task. During this phase, participants were explicitly instructed to observe their emotional reactions without judgment and to allow those reactions to arise and pass naturally, without attempting to alter or suppress them—core principles of the acceptance approach. Following each image, participants were asked to evaluate their emotional experience using the Self‐Assessment Manikin (SAM) scale (Bradley and Lang 1994) on separate 9‐point scales for valence and arousal (1 = very unpleasant/not arousing; 9 = very pleasant/highly arousing). This step both reinforced participants' attention to their emotional responses and familiarized them with the rating procedure used in the EEG task.

#### 2.3.2 Task and Design Experiment

Upon arrival at the laboratory, participants provided informed consent and completed a demographic form including age, gender, and years of education. They also confirmed having no auditory impairments and reported no history of neurological, psychiatric, or learning disabilities.

Following this preliminary screening, a 64‐channel EEG cap was fitted according to the international 10–10 system. To assess baseline and post‐task neural activity, participants completed a 10‐min resting‐state EEG recording (eyes closed) before and after the main experimental task. Participants then received both written and verbal instructions outlining the structure of the experimental task and the correct implementation of the acceptance strategy. The experiment was programmed and executed using E‐Prime 2.0 software (Version 2.0.10.356, Psychology Software Tools). The study used a within‐subjects design with two blocks: a Look condition, in which participants passively observed emotional words, and an Accept condition, in which they applied acceptance strategy during word presentation. Each block contained 120 words, including 40 neutral, 40 negative, and 40 taboo items, yielding a total of 240 unique trials. The stimuli were divided into two comparable sets and presented only once per participant. The assignment of stimulus sets to conditions, as well as the order of the two blocks, was counterbalanced across participants. Within each block, words were presented in a fully randomized order.

**FIGURE 1:**
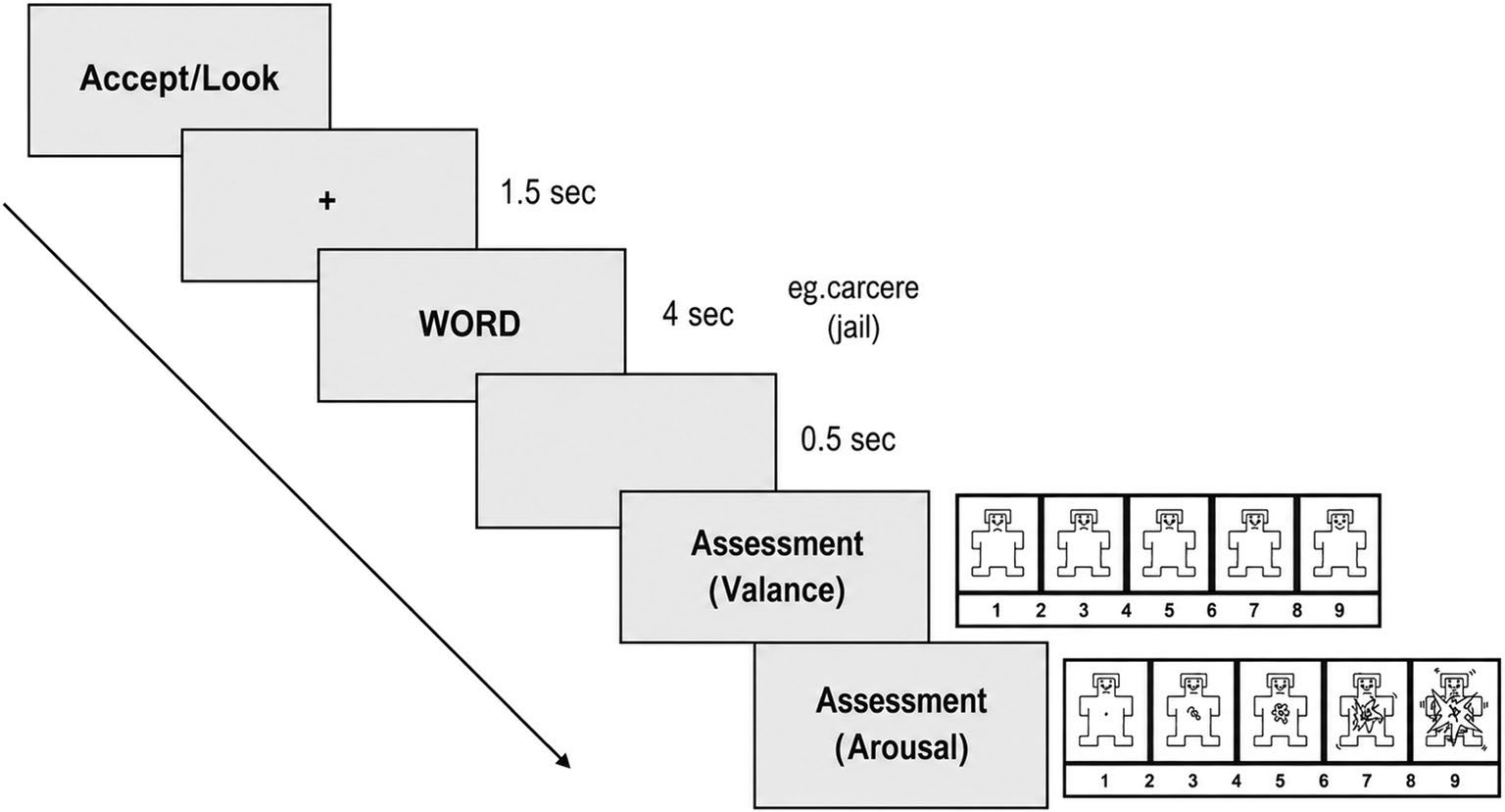
Experiment design.

Each trial began with a central fixation cross presented for 1.5 s, followed by a target word displayed for 4 s. After a 0.5‐s blank screen, participants rated their emotional experience in response to the word using the SAM (SAM; Bradley and Lang 1994) scale. Specifically, they provided valence and arousal ratings on separate 9‐point scales, where 1 indicated “very unpleasant” or “not arousing,” and 9 indicated “very pleasant” or “very arousing.” The trial ended with a 2‐s inter‐trial interval. Participants were encouraged to take breaks at regular intervals, approximately every 40 trials, to reduce cognitive fatigue and maintain concentration. The entire experimental session, including EEG cap preparation, instruction, training, and task performance, lasted approximately 150 min (See Figure 1).

### 2.4 EEG Data Acquisition

EEG recordings were conducted using the eegosport (ANT) system at a sampling rate of 1000 Hz using 64 Ag–AgCl scalp electrodes arranged in the standard 10/10 layout. The CPz electrode was used as the online reference, and electrode impedances were kept below 20 kΩ. Additionally, an electrooculogram (EOG) was obtained using electrodes positioned below the left eye. For signal processing, we used EEGLAB (Delorme and Makeig 2004) (version 2021.1) and FieldTrip toolbox (Oostenveld et al. 2011) (version fieldtrip‐20230913), together with custom scripts within the MATLAB environment (version 2021b, MathWorks Inc).

### 2.5 EEG Pre‐Processing

The mastoid electrodes were not included in the analysis. The EEG data were first subjected to a 0.1 Hz high‐pass filter, followed by an 80 Hz low‐pass filter using a second‐order Butterworth filter for both stages. The filtered continuous EEG signal was segmented into epochs extending from 1500 ms before to 4500 ms after stimulus onset. Noisy channels were interpolated using spherical interpolation (*M* = 0.77), and the data were re‐referenced to the average activity across all electrodes. An Independent Component Analysis (ICA) was performed using the AMICA algorithm (Palmer et al. 2008), and components associated with eye blinks or saccades were removed (*M* = 1.85 ± 0.40). To address potential rank‐deficiency issues, the number of independent components was calculated while considering both the number of interpolated channels and the use of the average reference. Additional artifact rejection steps included the removal of noisy epochs where the signal exceeded ±100 μV in any channel, with an average of 6.66% epochs discarded. Participants were excluded from the EEG analyses when more than 25% of their data were removed. Average ERPs were computed for each condition and electrode.

**FIGURE 2:**
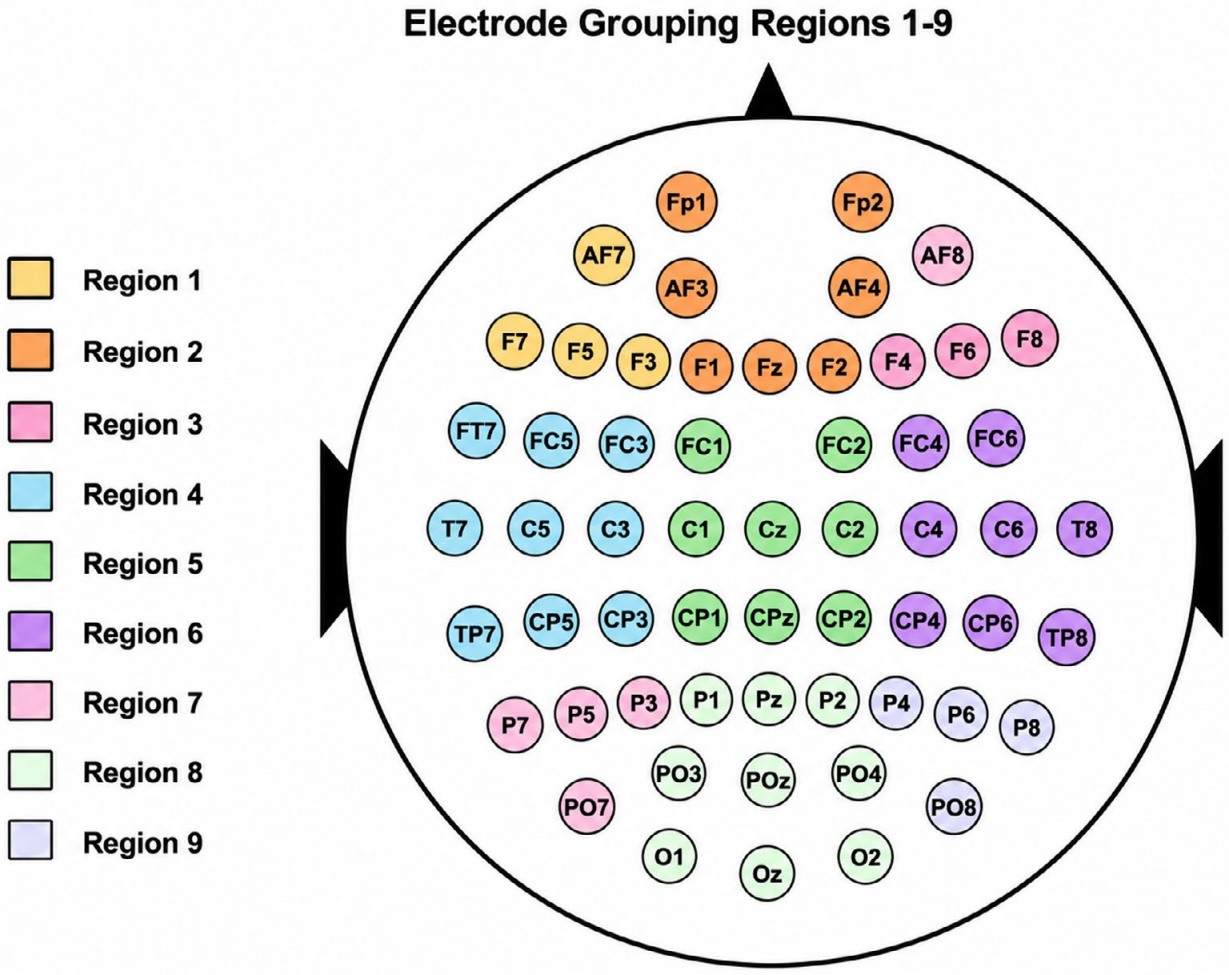
Electrode grouping layout region 1–9.

Previous studies have extensively described the spatio‐temporal dynamics of LPP modulations triggered by emotional words (e.g., Citron 2012). However, the specific impact of these modulations within our experimental setup was less clear. We used two approaches to choose the scalp electrodes and time points for analysis. First, to identify the most relevant channels and time windows, we conducted a cluster‐based permutation analysis (Maris and Oostenveld 2007). This analysis was aimed at comparing negative and neutral stimuli to pinpoint the temporal and spatial coordinates that would be most effective for later evaluations of emotion regulation strategies. The cluster‐based permutation was hence conducted on the same dataset used for classification and was implemented as an exploratory, data‐driven step performed prior to the machine learning analysis. We included all data from 0.4 to 1.5 s centered on stimulus onset, encompassing data from all electrodes. The analysis identified three significant clusters. Aggregation of these clusters helped in pinpointing key channels and timing for further study. Specifically, we focused on channels active for at least 50% of the cluster's duration and on samples in which at least 50% of the cluster's channels were involved. Based on these criteria, we selected a set of centro‐parietal electrodes (P5, PO5, PO3, P3, P1, CP3, Pz, POz, CP1, C3, CPz, C1, CP2, P2, and Cz), and a time window from 0.637 to 0.878 s for the classification decoding analysis. Crucially, both spatial and temporal features identified by this procedure align with traditional LPP coordinates, providing a standard electrophysiological framework for the decoding analysis. The corresponding set of electrodes was labeled as Region 10 and included as a data‐driven region of interest in the classification analysis (see Table 2 and Figures 1 and 2).

**TABLE 2:** Electrode groupings.

Region	Laterality	Region	Electrodes
Anterior	Left	1	F3, F5, F7, AF7
	Center	2	FP1, FPZ, FP2, AFZ, AF4, AF3, F1, FZ, F2
	Right	3	F4, F6, F8, AF8
Central	Left	4	FC3, FC5, FT7, T7, C3, C5, T7, CP3, CP5, TP7
	Center	5	FC1, FCZ, FC2, C1, CZ, C2, CP1, CPZ, CP2
	Right	6	FC4, FC6, FT6, C4, C6, T8, CP4, CP6, TP8
Posterior	Left	7	P3, P5, P7, PO7, PO5
	Center	8	P1, PZ, P2, PO3, POZ, PO4, O1, OZ, O2
	Right	9	P4, P6, P8, PO6, PO8
		10	P5, PO5, PO3, P3, P1, CP3, Pz, POz, CP1, C3, CPz, C1, CP2, P2, and Cz

**FIGURE 3:**
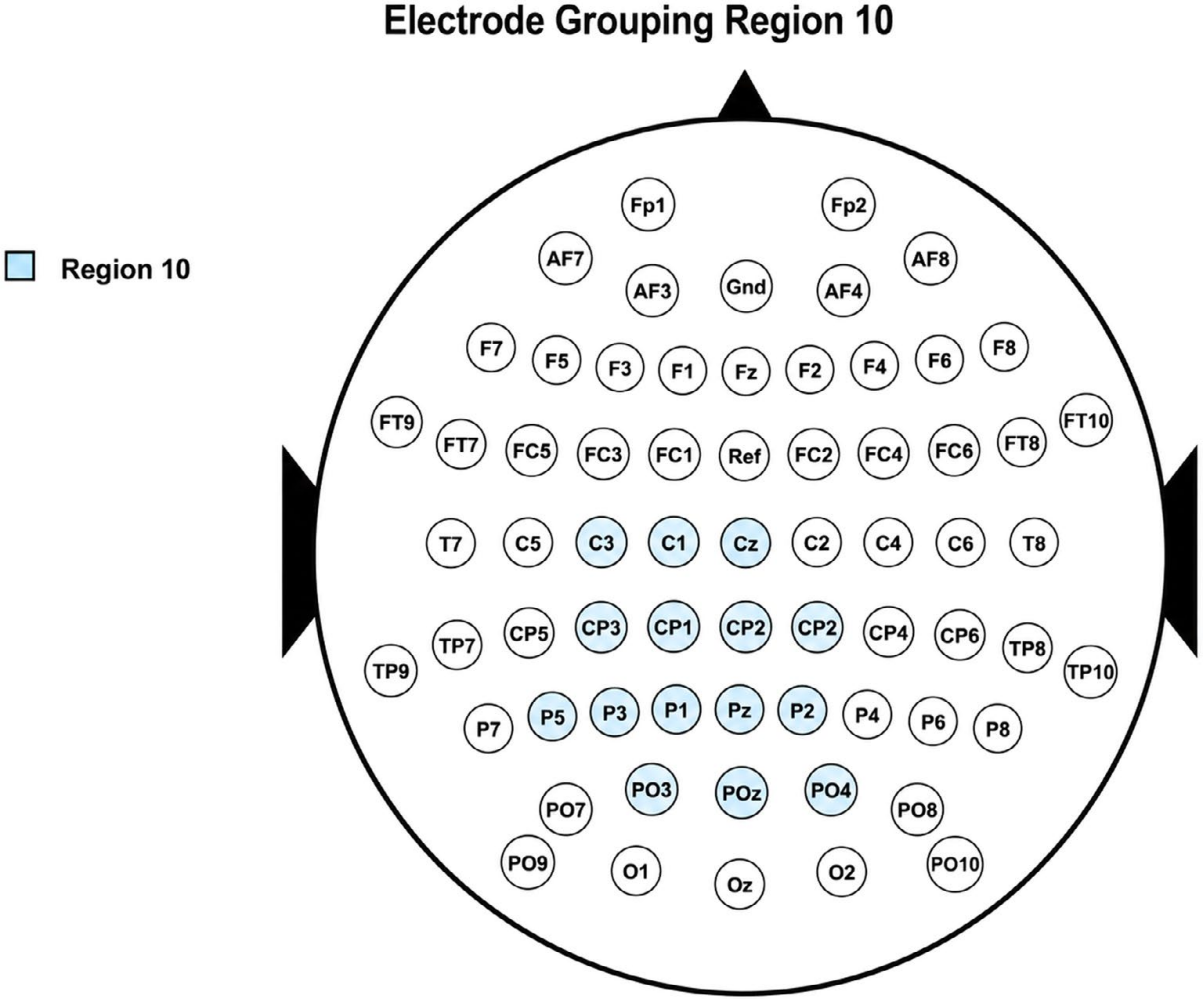
Electrode grouping layout region 10.

In addition to the data‐driven cluster‐based approach, we implemented a complementary region‐based segmentation strategy to examine the spatiotemporal dynamics of ERP activity more broadly across the scalp. This combination provided both a data‐driven identification of responsive sites and a theory‐informed examination of expected LPP patterns across anterior, central, and posterior regions. To this end, the scalp was systematically divided into three main areas—anterior, central, and posterior—and each of these was further subdivided into left, midline, and right regions. This yielded a total of nine distinct scalp regions (Regions 1–9; see Table 2 and Figure 3), allowing for a structured and symmetric analysis of ERP topography. For each region, we computed the average activity across all constituent electrodes, focusing on two time windows of interest: an early phase (450–649 ms) and a late phase (650–850 ms). These windows were selected based on visual inspection of grand‐averaged ERP waveforms across all conditions and were intended to capture the known temporal evolution of the Late Positive Potential (LPP). Although the selection was exploratory, it was informed by prior literature that consistently reports enhanced LPP activity within these intervals in response to emotionally salient stimuli. By analyzing both early and late phases, we aimed to capture potential shifts in the timing or intensity of emotional processing across conditions.

To further investigate the effect of strategy independently of stimulus type and the effect of regulation, we conducted a secondary analysis that collapsed across word categories. Specifically, we averaged brain activity across all trials and trained a classifier to distinguish between the Look and Accept conditions using features extracted from the same nine scalp regions and two time‐windows. This analysis enabled us to test for potential differences in global cortical activity patterns attributable solely to the use of the regulation strategy, independent of the emotional content of the stimuli.

Our decision to use both the cluster‐based and region‐based approaches reflects their complementary strengths. The cluster‐based permutation analysis offered a fine‐grained, data‐driven map of experiment‐specific effects, identifying the scalp regions and time points most sensitive to the contrast between negative and neutral words. This localized focus allowed us to hone in on well‐established LPP dynamics in a statistically rigorous way. In contrast, the region‐based approach offered a broader, hypothesis‐guided view of ERP activity across the entire scalp. By using geometrically defined regions and theoretically motivated time windows, we ensured that no potentially informative scalp areas were overlooked, including those outside of the data‐driven clusters. This dual approach enabled a deeper understanding of the temporal and spatial characteristics of neural responses to affective word categories and their modulation by emotion regulation strategies.

### 2.6 Behavioral Analyses

To examine participants' subjective emotional responses to the stimuli, we analyzed valence and arousal ratings using both omnibus and pairwise statistical tests. Our primary objective was to assess whether the three word categories—neutral, negative, and taboo—elicited distinct affective ratings and whether these effects were consistent across the Look and Accept conditions. We first conducted a repeated‐measures ANOVA with Condition (Look, Accept) and Word Category (Neutral, Negative, Taboo) as within‐subjects factors, separately for valence and arousal dimensions. Although this model allowed for the assessment of potential interaction effects between condition and stimulus type, no significant interaction emerged. Therefore, we proceeded with planned pairwise comparisons to directly test our hypotheses. Specifically, we performed a series of paired‐samples *t*‐tests within each condition, comparing the following contrasts: Neutral versus Taboo, Neutral versus Negative, and Negative versus Taboo. This approach enabled us to examine the relative emotional impact of the word categories in a way that mirrors the logic of the subsequent neural classification analyses, which also operate on pairwise distinctions between stimulus types.

### 2.7 Machine Learning Analysis

We applied SVM classification model inside NeuroMiner toolbox (NM) version 1.05 (Koutsouleris et al. 2018) in Matlab (v.2023). The objective of the model was to distinguish between neutral versus negative, neutral versus taboo, negative versus taboo word stimuli separately within both the “Look” and “Accept” conditions and to investigate the effect of strategy independent of stimuli type. The model was developed using a repeated‐nested double cross‐validation (CV) method. This method is crucial for preventing information leakage, reducing the risk of overfitting, and providing an unbiased estimation of the model's generalizability to novel data (Dwyer et al. 2018; Koutsouleris et al. 2016; Ruschhaupt et al. 2005). This cross‐validation structure included two nested k‐fold CV cycles: an inner cycle (CV1) where models are built, and a superordinate outer cycle (CV2) where they are tested for generalizability (Filzmoser et al. 2009). Each cycle consisted of 10 folds and underwent 10 permutations. Preprocessing in CV1 involved removing zero‐variance features and normalizing matrices to the mean (Inza et al. 2010). Missing values were addressed using a *k* = 7 nearest neighbor method for imputation (Troyanskaya et al. 2001). The data processed in CV1 was then fed into a linear, class‐weighted SVM algorithm (LIBSVM 3.1.2 L1‐Loss SVM) to construct a hyperplane for making predictions in the training and test sets. The optimal analysis pipeline identified was subsequently applied to each k‐fold and each permutation in the CV2 cycle. The classification of word stimuli (neutral, taboo; neutral, negative; negative, taboo) in both conditions was determined by a majority vote across all ensemble models. Permutation testing was conducted to establish statistical significance at an alpha level of 0.05 with 1000 permutations (Golland and Fischl 2003).

The performance metrics for the model included Balanced Accuracy (BAC), sensitivity, specificity, Positive Predictive Value (PPV), Negative Predictive Value (NPV), and the Area Under the Curve (AUC). BAC provides an overall measure of the model's effectiveness, expressed as a percentage, balancing its ability to correctly classify both classes (e.g., neutral and taboo stimuli), especially when there is class imbalance. Sensitivity (or True Positive Rate) is expressed as a percentage and reflects the model's ability to correctly identify instances of one class, such as accurately classifying taboo stimuli when they are indeed taboo. Specificity (or True Negative Rate), also expressed as a percentage, indicates the model's effectiveness in correctly identifying instances of the other class, such as accurately classifying neutral stimuli when they are indeed neutral, thereby avoiding incorrect classifications. PPV is expressed as a percentage and indicates the proportion of instances classified as taboo that are indeed taboo, demonstrating how reliable the model is when it predicts a taboo outcome. NPV, similarly expressed as a percentage, represents the proportion of instances classified as neutral that are indeed neutral, showing the reliability of the model when predicting a neutral outcome. Lastly, the AUC provides a comprehensive measure of the model's overall ability to distinguish between the neutral and taboo classes across all possible thresholds; it is typically expressed as a value between 0 and 1, rather than a percentage, with higher values indicating better overall performance.

The input features for classification were the averaged ERP amplitudes extracted from the previously defined spatiotemporal regions of interest. These included the nine anatomically defined scalp regions (Regions 1–9) and the data‐driven cluster‐based region (Region 10), across three key time windows: 450–649, 650–850, and 637–878 ms.

## 3 Results

### 3.1 Behavioral Results

#### 3.1.1 Word Categories in the Look Condition

Valence ratings indicated that neutral stimuli were rated as more positive than both taboo stimuli, *t* (34) = 5.79, *p* < 0.001, *M*diff = 0.92, and negative stimuli, *t* (34) = 9.92, *p* < 0.001, *M*diff = 1.93. Taboo stimuli were rated as more positive than negative stimuli, *t* (34) = −13.19, *p* < 0.001, *M*diff = −1.01. For arousal, both taboo, *t* (34) = −8.65, *p* < 0.001, *M*diff = −1.44, and negative stimuli, *t* (34) = −6.32, *p* < 0.001, *M*diff = −1.61, were rated as more arousing than neutral stimuli. There was no significant difference between negative and taboo stimuli, *t* (34) = 1.11, *p* = 0.275, *M*diff = 0.16.

#### 3.1.2 Word Categories in the Regulation Condition

Results indicated significant differences in valence across categories. Neutral stimuli were rated as more positive than both taboo stimuli, *t* (34) = 5.37, *p* < 0.001, *M*diff = 0.90, and negative stimuli, *t* (34) = 8.31, *p* < 0.001, *M*diff = 1.88. Taboo stimuli were rated as more positive than negative stimuli, *t* (34) = −7.80, *p* < 0.001, *M*diff = −0.98. For arousal, negative stimuli were rated as more arousing than neutral stimuli, *t* (34) = −6.55, *p* < 0.001, *M*diff = −1.52, and there was no significant difference between negative and taboo stimuli, *t* (34) = 1.12, *p* = 0.27, *M*diff = 0.15. See further details in Tables 3 and 4.

**TABLE 3:** Behavioral result.

Condition	Variable	Comparison	*t*	df	*p*	Mean difference	95% CI Lower	95% CI Upper
Look	Valence	Neutral versus Taboo	5.79	34	< 0.001	0.92	0.6	1.24
Look	Arousal	Neutral versus Taboo	−8.65	34	< 0.001	−1.44	−1.78	−1.1
Look	Valence	Neutral versus Negative	9.92	34	< 0.001	1.93	1.54	2.33
Look	Arousal	Neutral versus Negative	−6.32	34	< 0.001	−1.61	−2.12	−1.09
Look	Valence	Negative versus Taboo	−13.19	34	< 0.001	−1.01	−1.17	−0.85
Look	Arousal	Negative versus Taboo	1.11	34	0.28	0.16	−0.13	0.46
Regulate	Valence	Neutral versus Taboo	5.37	34	< 0.001	0.9	0.56	1.24
Regulate	Arousal	Neutral versus Taboo	−9.06	34	< 0.001	−1.37	−1.68	−1.06
Regulate	Valence	Neutral versus Negative	8.31	34	< 0.001	1.88	1.42	2.34
Regulate	Arousal	Neutral versus Negative	−6.55	34	< 0.001	−1.52	−2	−1.05
Regulate	Valence	Negative versus Taboo	−7.80	34	< 0.001	−0.98	−1.24	−0.73
Regulate	Arousal	Negative versus Taboo	1.12	34	0.27	0.15	−0.13	0.43

Abbreviations: CI: confidence interval; df: Degrees of freedom.

**TABLE 4:** Performance of SVM model across different conditions and stimulus types.

Condition	Stimulus comparison	B A	Sensitivity	Specificity	PPV	NPV	AUC	95% CI	*p*	Cohen's *d*
Look	Neutral versus Taboo	70.00%	74.30%	65.70%	68.40%	71.90%	0.8	[54.8, 85.2]	0.01	1.19
	Neutral versus Negative	54.30%	57.10%	51.40%	54.10%	54.50%	0.6	[37.8, 70.8]	0.031	0.36
	Negative versus Taboo	65.70%	74.30%	57.10%	63.40%	69.00%	0.7	[50.0, 81.4]	0.01	0.74
Accept	Neutral versus Taboo	68.60%	68.60%	68.60%	68.60%	68.60%	0.7	[53.2, 84.0]	0.01	0.74
	Neutral versus Negative	62.90%	65.70%	60.00%	62.20%	63.60%	0.6	[46.9, 78.9]	0.01	0.36
	Negative versus Taboo	57.10%	51.40%	62.90%	56.40%	57.70%	0.6	[40.7, 73.5]	0.01	0.36
Accept versus Look	Independent of Stimuli type	34.30%	24.30%	45.70%	36.20%	37.20%	0.22	[18.8, 49.8]		

*Note:* Effect sizes (Cohen's *d*) were derived from AUC values to provide a standardized index of pattern separability across decoding contrasts.

Abbreviations: AUC = area under curve; NPV = negative predictive value; PPV = positive predictive value.

### 3.2 EEG Results

#### 3.2.1 Electrophysiological Characterization (Grand‐Average ERPs)

**FIGURE 4:**
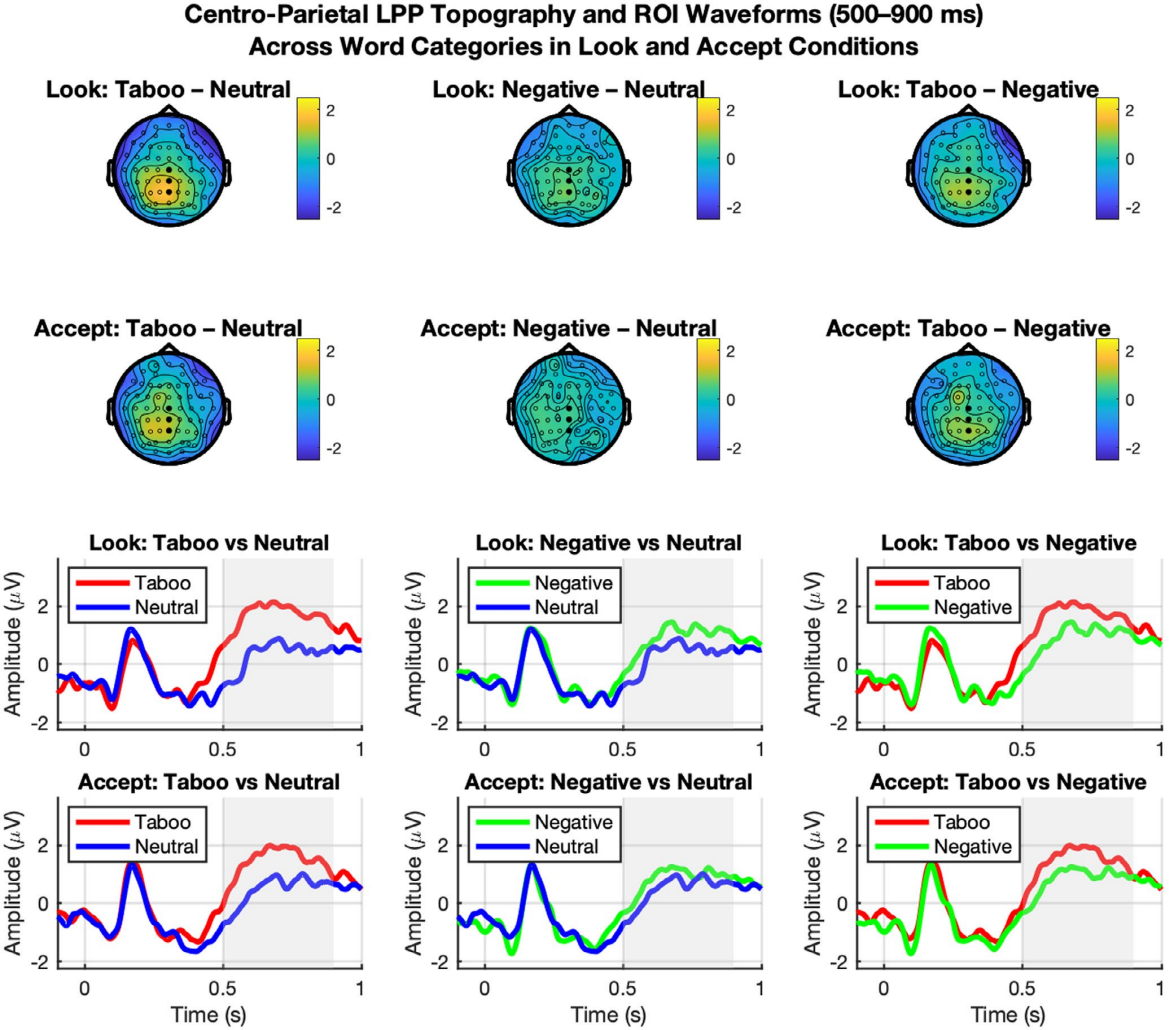
Grand‐average topographic maps and ERP waveforms. The top two rows display the topographic distribution of word‐category contrasts (Taboo–Neutral, Negative–Neutral, and Taboo–Negative) for both Look (top) and Accept (bottom) conditions. Highlighted electrodes denote Region 10, the centro‐parietal cluster where significant differences were identified. The bottom two rows show the corresponding grand‐average ERP waveforms for these contrasts, averaged across all electrodes in Region 10. Shaded gray areas represent the 637–878 ms temporal window used for the topographic maps, where significant differences between categories were observed (*p* < 0.05).

To provide a conventional characterization of the neural response to emotional words, we first examined the grand‐average ERP waveforms and topographic distributions. As illustrated in Figure 4, both negative and taboo words elicited a more positive‐going deflection compared to neutral words over posterior‐parietal sites, consistent with the morphology of the Late Positive Potential (LPP). This effect was visible in both the Look and Accept conditions. The topographic maps further confirm that the differences between word categories were most pronounced in the central‐parietal region, confirming that our data‐driven Region 10 captures the core of the electrophysiological activity typically associated with affective word processing (see also Figure 4).

#### 3.2.2 Word Categories Perception

**FIGURE 5:**
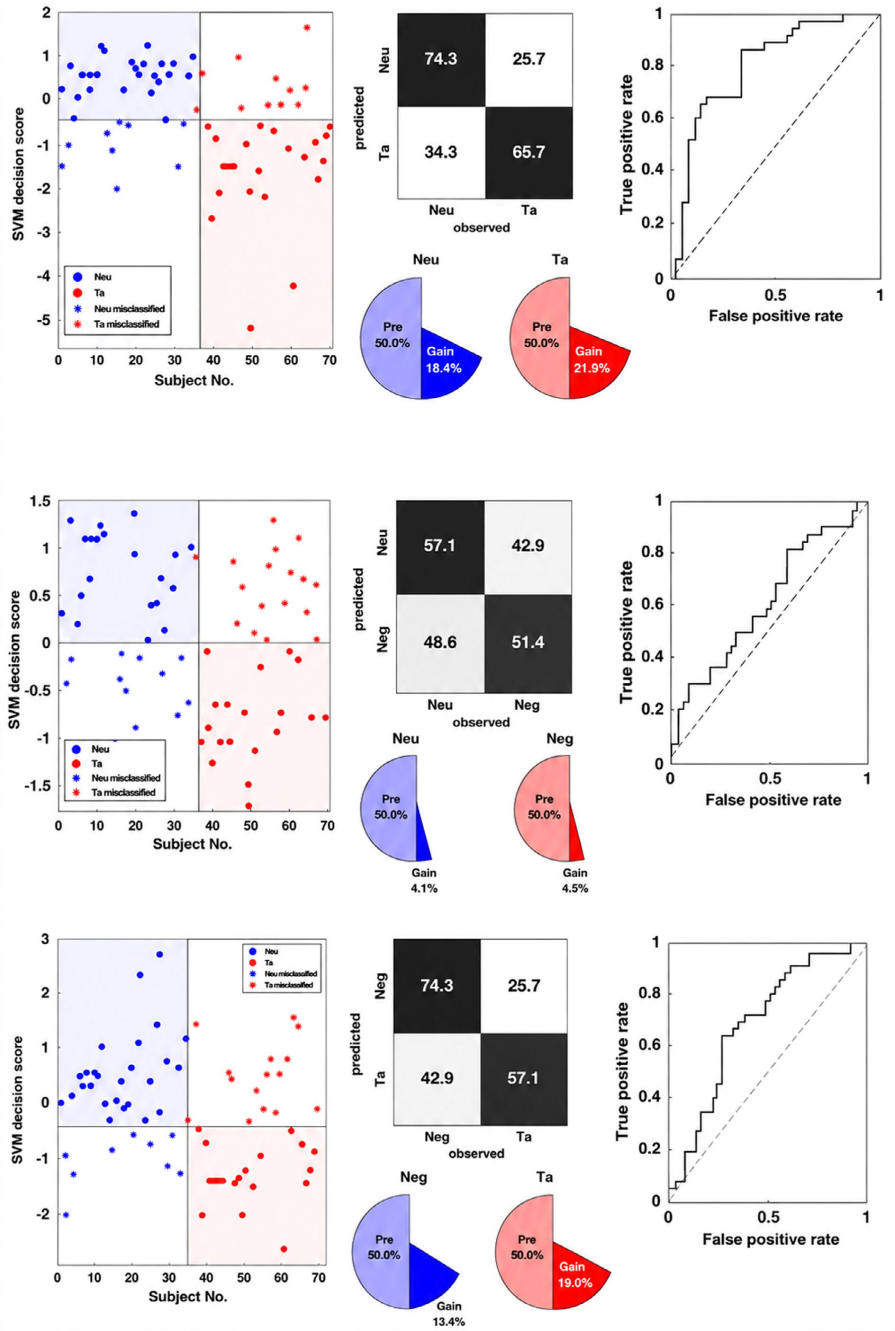
SVM classification performance in the Look condition. Top figure: SVM decision scores distinguishing Neutral (Neu) from ‘Taboo (Ta)’ stimuli, with the confusion matrix and Receiver Operating Characteristic (ROC) curve displayed on the right. Middle figure: SVM decision scores for Neutral versus Negative stimuli, alongside the confusion matrix and ROC curve. Bottom figure: SVM decision scores for Negative and Taboo stimuli, with the corresponding confusion matrix and ROC curve.

**FIGURE 6:**
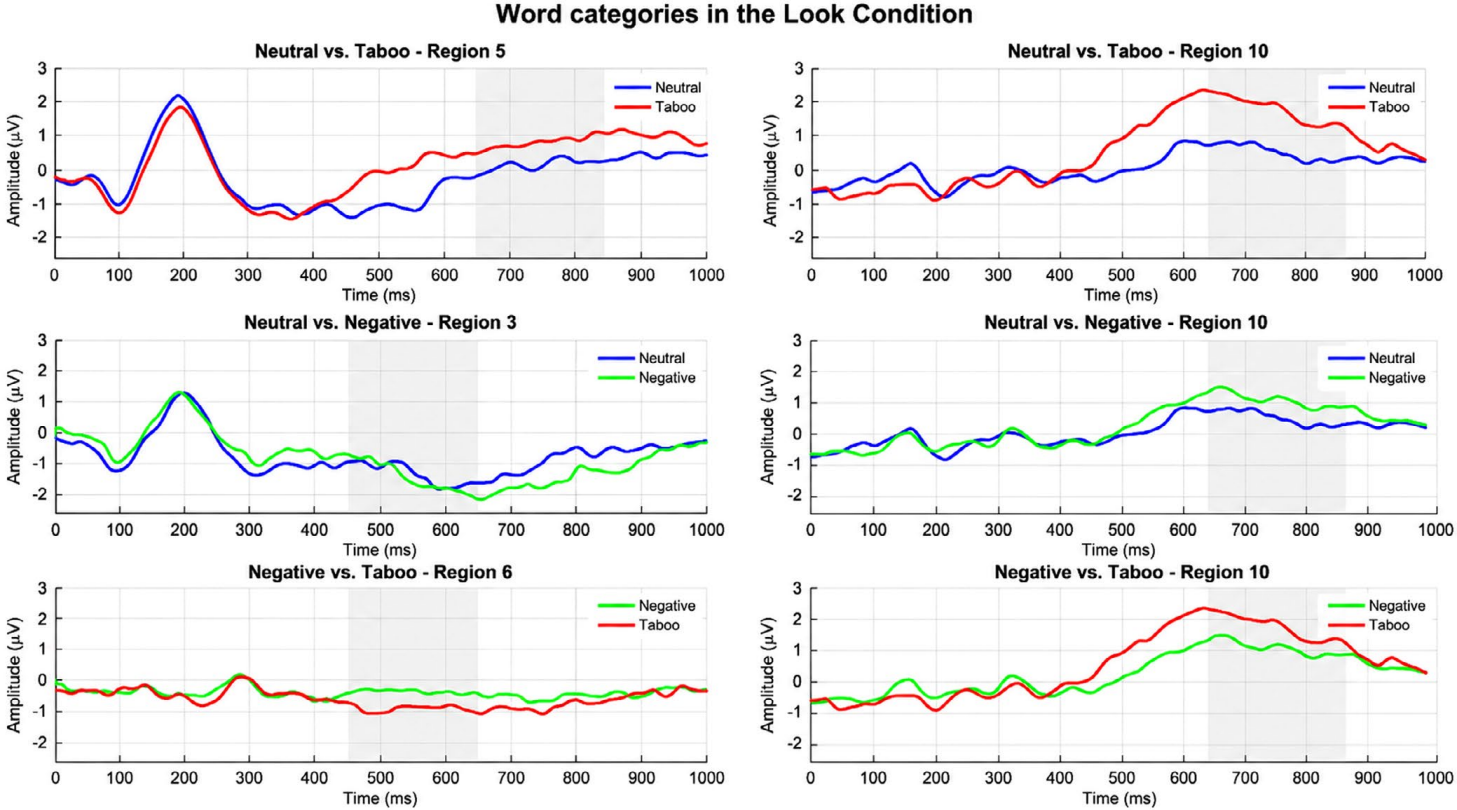
ERP plot of responses to word categories in the Look Condition. Top: Neutral versus Taboo, Middle: Neutral versus Negative, Bottom: Negative versus Taboo.

We assessed whether ERP patterns allowed decoding between word categories using multivariate pattern analysis. While conventional univariate ERP analyses are commonly used to characterize emotional word processing, the present study focused on multivariate decoding to assess whether distributed spatiotemporal ERP patterns discriminate between conditions beyond mean amplitude differences at individual electrodes. Given the high dimensionality and low signal‐to‐noise ratio of ERP data, decoding performance was interpreted in conjunction with effect size estimates rather than relying on accuracy values alone. The support vector machine model significantly distinguished between neutral and taboo stimuli with high accuracy and a large effect size, BAC = 70.0%, AUC = 0.80, *p* = 0.01, *d* = 1.19. This classification was primarily driven by Region 10 (central‐parietal‐occipital) and Region 5 (central scalp) between 637 and 878 ms. Classification of neutral versus negative stimuli yielded a significant but smaller effect, BAC = 54.3%, AUC = 0.60, *p* = 0.0316, *d* = 0.36, supported by Regions 10 and 3 (anterior right) during the 450–649 ms window. Finally, the model differentiated negative from taboo stimuli with a medium‐to‐large effect size, BAC = 65.7%, AUC = 0.69, *p* = 0.01, *d* = 0.74, specifically utilizing Region 6 (central right) and Region 10 within the 450–649 ms window (see Figures 5 and 6).

#### 3.2.3 Effect of Regulation

**FIGURE 7:**
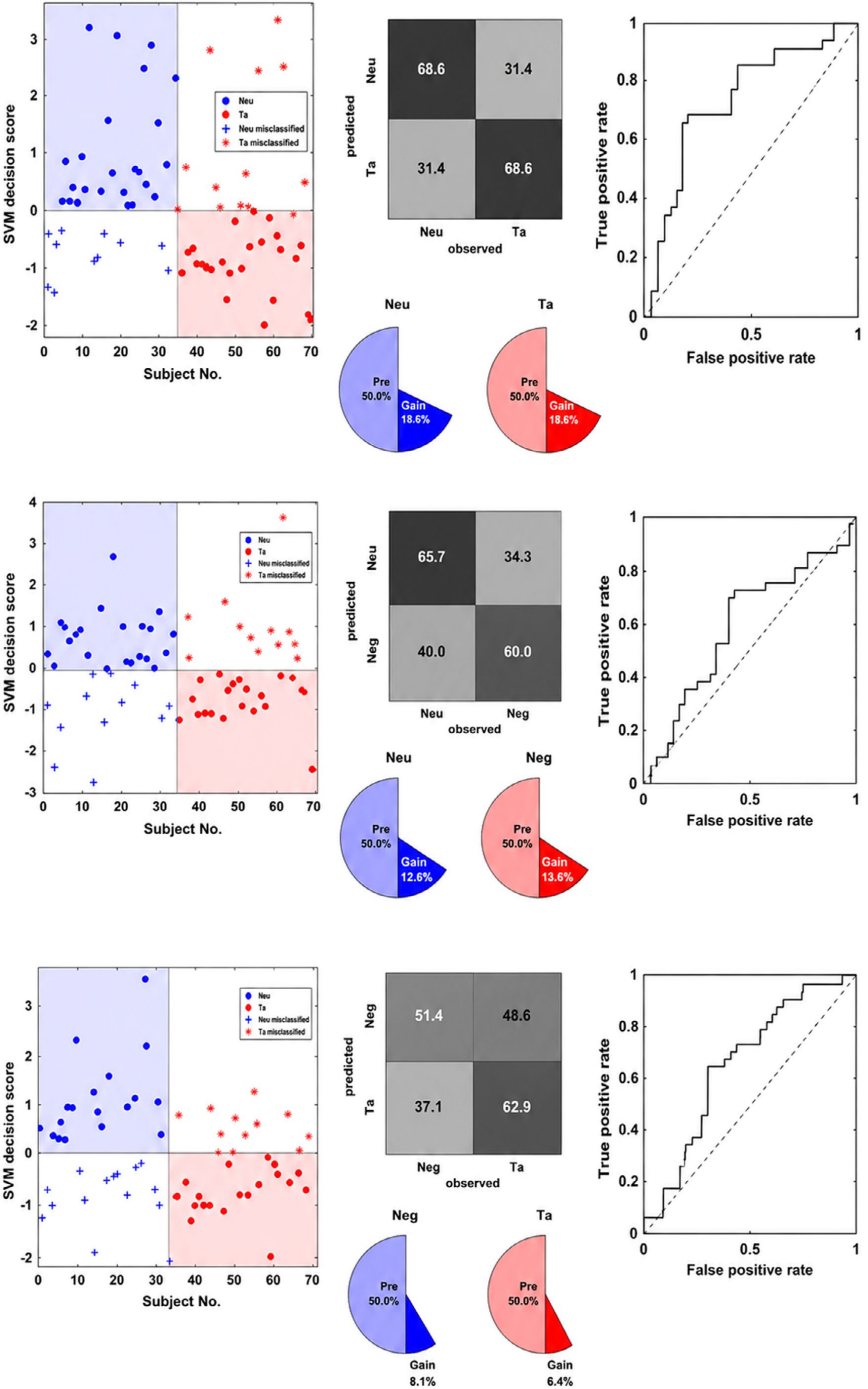
SVM classification performance in the Accept condition. Top panel: SVM decision scores differentiating ‘Neutral (Neu)’ from ‘Taboo (Ta)’ stimuli, with the confusion matrix and Receiver Operating Characteristic (ROC) curve on the right. Middle panel: SVM decision scores for ‘Neutral (Neu)’ versus Negative (Neg) stimuli, alongside the confusion matrix and ROC curve. Bottom panel: SVM decision scores for ‘Negative (Neg)’ and Taboo (Ta) stimuli, with the corresponding confusion matrix and ROC curve.

**FIGURE 8:**
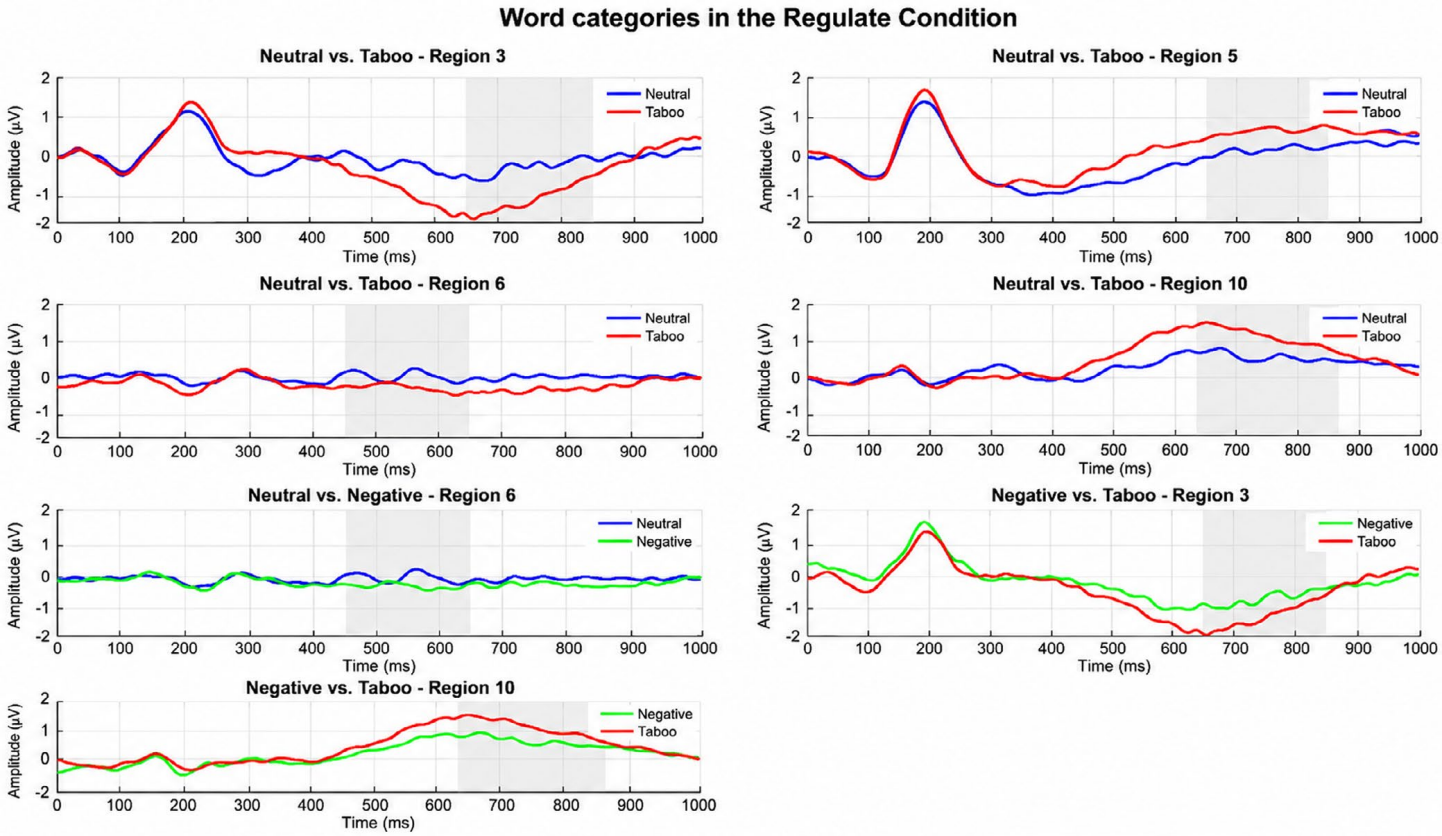
ERP plot of responses to word categories in the Regulate Condition. Top and second rows: Neutral versus Taboo. Third row left: Neutral versus Negative. Third row right and bottom row: Negative versus Taboo.

In the Accept condition, the model significantly distinguished between neutral and taboo stimuli with a large effect size, BAC = 68.6%, AUC = 0.74, *p* = 0.01, *d* = 0.91. This performance was driven by central‐right features (450–649 ms), frontal‐right regions (650–850 ms), and Region 10 in the central‐parietal‐occipital area (637–878 ms). Classification between neutral and negative words yielded BAC = 62.9%, AUC = 0.58, *p* = 0.01, *d* = 0.28, primarily localized to Region 6 in the central‐right area (450–649 ms). Finally, the model differentiated negative versus taboo words with BAC = 57.1%, AUC = 0.61, *p* = 0.01, *d* = 0.39, with significant contributions from Region 3 (650–850 ms) and Region 10 (see Table 1 and Figures 7 and 8).

#### 3.2.4 Effect of Strategy

To examine whether acceptance (emotion regulation strategy) itself produced a distinct neural signature (effect of strategy), we trained a SVM classifier to differentiate between the Look and Accept conditions regardless of stimulus type. This analysis tested our third hypothesis: that acceptance, being a bottom‐up strategy, would not engage cortical activity.

**FIGURE 9:**
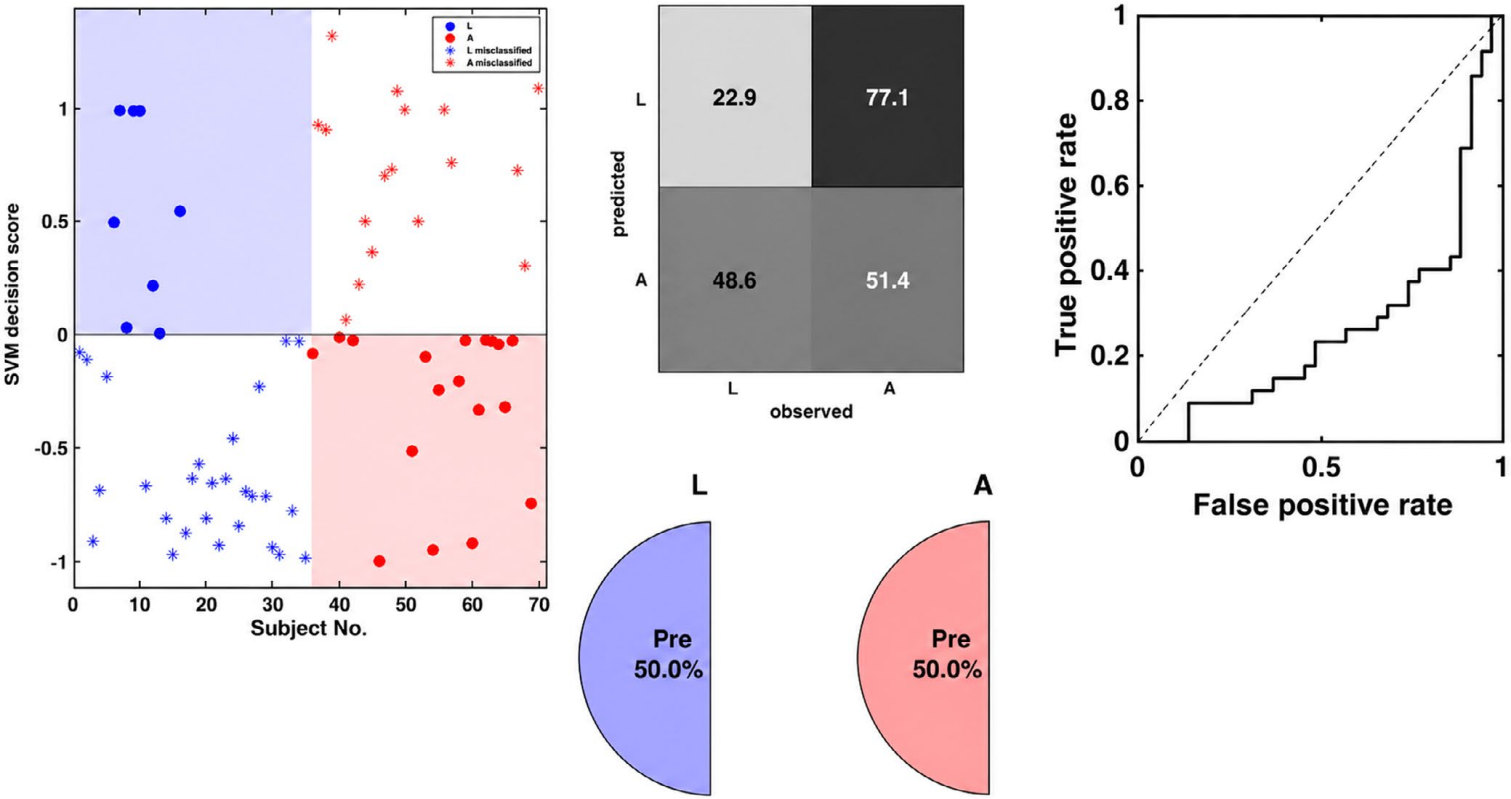
SVM classification performance in the Accept and Look condition. Top panel: SVM decision scores differentiating Accept (A) from Look (L) condition, with the confusion matrix and Receiver Operating Characteristic (ROC) curve on the right.

The SVM model showed poor classification performance, with a balanced accuracy (BAC) of only 37.1% and the AUC was 0.26, well below chance level (see Table 4 Figure 9).

## 4 Discussion

The present study examined whether distributed ERP patterns allow reliable decoding of neutral, negative, and taboo words. A second aim was to assess whether comparable classification could be achieved in the emotion regulation condition (Accept), where participants engaged in acceptance‐based regulation. Lastly, we sought to examine whether acceptance (emotion regulation strategy) could be detected at the cortical level via EEG by attempting to distinguish the Accept condition from the Look condition.

Behavioral ratings confirmed the expected affective differentiation among categories. In the Look condition, neutral words were rated higher in valence than both taboo and negative words, with negative words rated lowest. Arousal ratings indicated higher arousal for taboo and negative relative to neutral words, with no reliable arousal difference between taboo and negative words. These patterns were replicated—albeit numerically attenuated—in the Accept condition, supporting the intended affective separation of the stimulus categories.

At the neural level, SVM revealed reliable decoding of word categories. During passive viewing, the classifier achieved its highest balanced accuracy (BAC = 70.0%) in distinguishing between neutral and taboo words. This classification relied on signals from the central‐parietal‐occipital region (Region 10) in the 637–878 ms window, and the central region (Region 5) in the 650–850 ms window, consistent with the known temporal and spatial distribution of the LPP (Schupp et al. 2000; Citron 2012; Hajcak et al. 2010). Performance was also above chance for negative versus taboo (BAC = 65.7%) and neutral versus negative (BAC = 54.3%) contrasts, with informative features drawn from both early (450–649 ms) and later (650–850 ms) LPP time windows, involving anterior and central‐right regions (Ito et al. 1998; Fields and Kuperberg 2012; Delaney‐Busch et al. 2016).

These results confirmed that word categories differ in their spatiotemporal ERP patterns, particularly during the LPP, a component sensitive to both valence and motivational salience (Citron 2012; Hajcak et al. 2010; Hinojosa et al. 2010). Notably, the distinctiveness of taboo words—even relative to other negative stimuli—suggests that their processing recruits additional dimensions beyond valence or arousal, perhaps related to their social salience and norm violation potential (Donahoo and Lai 2020; Sendek et al. 2022).

Decoding remained above chance in the Accept condition, indicating that category‐specific ERP patterns were preserved under acceptance‐based regulation. Although performance was slightly reduced relative to Look, this attenuation is broadly consistent with prior work showing that regulation instructions can dampen neural differentiation to affective stimuli (Thiruchselvam et al. 2011; Qi et al. 2017). Interestingly, the right frontal region emerged more prominently during the Accept condition, particularly in the 650–850 ms window. This area has been implicated in cognitive evaluation and contextual reappraisal of emotional content (Hajcak et al. 2010), suggesting that even acceptance—typically considered a bottom‐up process—may engage evaluative processes under certain conditions. Although the classifier was unable to distinguish directly between the Look and Accept conditions, its ability to separate emotional word categories varied across these strategies. This raises the possibility that acceptance may exert its influence not by generating a unique neural signature per se, but by altering how emotional content is processed. In other words, the modulation of neural responses to emotional stimuli under the Accept condition—particularly in the differentiation between taboo and neutral or negative content—might serve as an indirect “signature” of successful engagement with the strategy.

Increased decoding accuracy in the taboo versus neutral contrast persisted in both Look and Accept conditions. This likely reflects the enhanced LPP responses to socially and emotionally salient words (Fields and Kuperberg 2012; Herbert et al. 2008; Carretié et al. 2008), particularly in central‐parietal and right‐lateralized scalp regions. The fact that taboo and negative words were matched in arousal, yet differed in neural activation, indicates that tabooness introduces a dimension of processing not fully captured by traditional affective dimensions such as valence and arousal (Sulpizio et al. 2024). A particularly intriguing finding concerns the distinct neural processing of taboo words, which elicited stronger and more discriminable ERP responses than negative words. This effect is notable because taboo words were rated higher in valence than negative words (4.25 ± 1.03 vs. 2.19 ± 0.37) yet they still triggered more robust neural signatures despite being matched for arousal. Across both Look and Accept conditions, decoding was stronger for taboo than neutral words, consistent with enhanced late evaluative processing for socially salient language. Critically, this graded ERP response (Taboo > Negative > Neutral) cannot be explained by emotional valence or arousal alone and is unlikely to reflect emotion suppression, as participants were never instructed to inhibit responses and the results showed sustained centro‐parietal positivities rather than typical control‐related mechanisms. Instead, “tabooness” serves as a cognitive‐affective construct that captures elements of attention capture, cognitive conflict, and social norm violation (Donahoo and Lai 2020; Gisladottir et al. 2015, 2018). This pattern aligns with the interpretation that taboo words index a mandatory appraisal of socially regulated meaning and moral salience (Donahoo and Lai 2020; Sulpizio et al. 2024). In this framework, while negative valence accounts for the presence of LPP/LPC activity in both categories, the specific “tabooness” reflects the additional processing of socially constrained, norm‐violating meaning.

Finally, we assessed whether the Accept strategy could be differentiated from the Look condition independent of stimulus type. The comparison between the Look and Accept conditions, the SVM model yielded a balanced accuracy of 37.1% and an AUC of 0.26. While we initially hypothesized that acceptance‐based strategies might produce negligible cortical signatures, which would typically result in a chance‐level AUC of approximately 0.50, the observed result is systematically below chance. This suggests a systematic misassignment, where the neural patterns associated with the Accept condition are consistently treated as the opposite of the Look condition. Accordingly, the present findings do not allow us to draw strong conclusions regarding the underlying neural substrates. Also, this result may reflect methodological factors such as limited statistical power, variability in participants' engagement with the acceptance strategy, or the low signal‐to‐noise ratio inherent to ERP data. Future studies combining EEG with other methodologies or incorporating more stringent manipulation checks will be important to directly evaluate this possibility.

In sum, our findings provide compelling evidence that emotional word categories can be reliably distinguished based on neural activity during passive viewing. This neural differentiation remains detectable, albeit attenuated, when participants engage in acceptance‐based emotion regulation. Furthermore, the acceptance strategy itself does not produce a discernible cortical signature measurable via EEG, supporting the notion that it primarily engages subcortical, bottom‐up processes. Finally, the results reveal that taboo words elicit distinct neural responses that cannot be fully explained by conventional affective dimensions such as valence and arousal, suggesting that their social and contextual salience plays a critical role in how they are processed. These findings demonstrate the utility of machine learning methods for decoding affective brain states and highlight the importance of considering linguistic‐social context, not just emotional valence, when examining the neural basis of emotional word processing.

## 5 Conclusions and Limitations

The present study explored the potential of machine learning techniques to decode affective word categories—neutral, negative, and taboo—from EEG signals. Our findings demonstrate that SVM classifiers can successfully distinguish these categories based on ERP activity, with performance exceeding chance levels in both passive observation (Look) and emotion regulation (Accept) conditions. The most informative neural features were located in the central‐parietal‐occipital and anterior right scalp regions, particularly within time windows associated with the LPP. These findings are consistent with prior literature on the spatiotemporal dynamics of emotional word processing. Although classification accuracy was slightly reduced during the Accept condition, it remained statistically significant. This suggests that acceptance‐based emotion regulation attenuates—but does not eliminate—the neural distinctions among affective word categories. Notably, taboo words consistently elicited stronger ERP responses and yielded the highest classification accuracy, even when their valence ratings were higher than those of negative words. This supports the interpretation that taboo words engage unique neural mechanisms, likely related to their social salience, cultural meaning, and norm‐violating character, which transcend basic affective dimensions such as valence and arousal. However, it should be noted that the present study used Italian word stimuli, and the norms for taboo words may vary across languages and cultures. Therefore, these findings should be interpreted carefully when generalizing beyond the Italian linguistic and cultural context. Future research should examine whether similar neural decoding patterns emerge in other languages or cultural settings.

Despite the robustness of our findings, several limitations warrant consideration. First, the exclusive use of written word stimuli may limit the generalizability of our results to broader emotional contexts. Emotional words differ in their processing demands compared to non‐verbal or multimodal stimuli (e.g., images, sounds, facial expressions). However, we chose word stimuli specifically to isolate linguistic emotional processing and to maintain experimental control over lexical variables. To strengthen ecological validity, future studies could expand the paradigm to include pictorial or audiovisual stimuli and compare neural decoding across modalities. Second, while decoding methods such as SVM are well‐suited to detect distributed neural patterns, they are not designed to localize brain activity with the anatomical precision of traditional source‐based analyses. Nevertheless, our study combined MVPA with a careful selection of time windows and scalp regions based on known ERP topographies (e.g., the LPP), allowing us to draw meaningful inferences about the timing and distribution of affect‐related neural signals. Future research may integrate decoding approaches with source localization techniques or combine EEG with fMRI to obtain a more complete picture of the neural generators involved. Third, while participants received training in the acceptance strategy and reported valence and arousal after each word, we did not collect direct behavioral measures assessing the successful application of acceptance during the main EEG task. As a result, we cannot determine the extent to which participants consistently or effectively engaged with the regulation strategy throughout the experiment. This limitation is particularly relevant for the interpretation of the null decoding results observed for the Look versus Accept contrast, as insufficient or variable engagement with the strategy could have contributed to the absence of reliable neural differentiation. Finally, a methodological asymmetry in feature selection should be noted. While the taboo versus negative contrast is strictly orthogonal, the selection of features based on the negative‐neutral contrast introduces a degree of circularity for decoding analyses involving the neutral category. Hence, the results for neutral‐involved contrasts should be interpreted with caution.

Future studies may include explicit manipulation checks, such as perceived‐success ratings or post‐block self‐reports, to validate engagement and strengthen the interpretation of neural regulation effects. In conclusion, this study demonstrates that machine learning applied to EEG data can decode subtle differences in emotional word processing, even under conditions of emotion regulation. It also underscores the distinctive status of taboo words as a neurocognitively and socially salient category, revealing complex interactions between emotion, language, and regulation strategies. These findings open the door to further exploration of how emotionally and socially charged language is represented in the brain, and how such representations are modulated in real‐time by internal control processes.

## Author Contributions

**Michele Scaltritti:** supervision, writing – review and editing, methodology. **Parisa Ahmadi Ghomroudi:** writing – original draft, formal analysis, data curation, visualization. **Remo Job:** writing – review and editing. **Peera Wongupparaj:** writing – review and editing. **Atefeh Jalali:** formal analysis, visualization. **Bianca Monachesi:** writing – review and editing. **Alessandro Grecucci:** methodology, conceptualization, supervision, formal analysis, writing – review and editing.

## Conflicts of Interest

The authors declare no conflicts of interest.

## Data Availability

The preprocessed EEG data supporting the findings of this study are available in the Open Science Framework (OSF) repository: https://osf.io/vfr72/?view_only=0085250ff7d44a1ea6ea73fad4f3d4a5.
